# Genome-wide RAD sequencing data provide unprecedented resolution of the phylogeny of temperate bamboos (Poaceae: Bambusoideae)

**DOI:** 10.1038/s41598-017-11367-x

**Published:** 2017-09-14

**Authors:** Xueqin Wang, Xiaying Ye, Lei Zhao, Dezhu Li, Zhenhua Guo, Huifu Zhuang

**Affiliations:** 1 Plant Germplasm and Genomics Center, Germplasm Bank of Wild Species, Kunming Institute of Botany, Chinese Academy of Sciences, Kunming, 650201 China; 2 College of Life Science and Agronomy, Zhoukou Normal University, Zhoukou, 466001 China; 3 Kunming College of Life Sciences, University of Chinese Academy of Sciences, Kunming, 650201 China; 4 University of Chinese Academy of Sciences, Beijing, 100049 China; 5 Key Laboratory of Biodiversity and Biogeography, Kunming Institute of Botany, Chinese Academy of Sciences, Kunming, 650201 China; 6 Key Laboratory of Economic Plants and Biotechnology, Yunnan Key Laboratory for Wild Plant Resources, Kunming Institute of Botany, Chinese Academy of Sciences, Kunming, 650201 China

## Abstract

The temperate bamboos (tribe Arundinarieae, Poaceae) are strongly supported as monophyly in recent molecular studies, but taxonomic delineation and phylogenetic relationships within the tribe lack resolution. Here, we sampled 39 species (36 temperate bamboos and 3 outgroups) for restriction-site associated DNA sequencing (RAD-seq) with an emphasis on *Phyllostachys* clade and related clades. Using the largest data matrix for the bamboos to date, we were able to infer phylogenetic relationships with unparalleled resolution. The *Phyllostachys*, *Shibataea*, and *Arundinaria* clades defined from plastid phylogeny, were not supported as monophyletic group. However, the RAD-seq phylogeny largely agreed with the morphology-based taxonomy, with two clades having leptomorph rhizomes strongly supported as monophyletic group. We also explored two approaches, BWA-GATK (a mapping system) and Stacks (a grouping system), for differences in SNP calling and phylogeny inference. For the same level of missing data, the BWA-GATK pipeline produced much more SNPs in comparison with Stacks. Phylogenetic analyses of the largest data matrices from both pipelines, using concatenation and coalescent methods provided similar tree topologies, despite the presence of missing data. Our study demonstrates the utility of RAD-seq data for elucidating phylogenetic relationships between genera and higher taxonomic levels in this important but phylogenetically challenging group.

## Introduction

The temperate bamboos (tribe Arundinarieae, Bambusoideae, Poaceae) are a clade of diverse taxa containing 32 genera and about 600 species^1–4^. Bamboos in this tribe have considerable ecological and economic value as most of species are major components of the subtropical and temperate forests in eastern and southeastern Asia. Many bamboo species are important sources of food, pulp manufacture, and materials for housing construction and artwork, such as Moso bamboo (*Phyllostachys edulis*)^5^. With highly diversified morphology and lack of flowering characters due to long vegetative periods, this tribe is notorious for the complicated taxonomy^1, 6^.

Although unequivocal sets of characters for classifying species and genera have not been identified, monophyly of temperate bamboos has been strongly supported in many molecular studies^7–12^. According to biogeographic analyses^13^, Arundinarieae diversified during the middle to late Miocene, and followed by a rapid radiation especially within the clades containing largest genera and species. Such recent origin might make the temperate bamboos undergo very little molecular variation^14^ and result in the intricate phylogenetic relationships within Arundinarieae. Based on broad sampling and eight non-coding plastid regions, Zeng *et al*.^15^ divided it into ten major lineages, and confirmed that most genera within this tribe were highly heterogeneous and incongruent with current taxonomic circumscriptions. Subsequently, two additional clades were recovered in Yang *et al*.^16^ and Zhang *et al*.^13^, thus twelve lineages in all were currently recognized in Arundinarieae based on plastid phylogeny. The evolutionary relationships among lineages were almost resolved but within lineages the resolution remained low^12, 17^, mainly due to the extremely slow molecular evolutionary rate of plastid DNA^15^. While in phylogeny based on nuclear DNA marker *GBSSI* ^18^, 13 lineages were resolved and incongruence was revealed between the plastid and nuclear trees, indicating different evolutionary trajectories. Moreover, the nuclear tree provided a poorly resolved phylogenetic relationship within Arundinarieae^16, 19^ because of insufficient informative characters, and more nuclear DNA markers were suggested to be needed to infer the evolutionary history of it.

Next-generation sequencing has recently been used to address evolutionary problems in the Bambusoideae^17, 20, 21^. Ma *et al*.^17^ and Attigala *et al*.^21^ used plastid genome sequencing to resolve the phylogenetic relationships in Arundinarieae and obtained robust relationships among the major clades. However, studies of Arundinarieae employing next-generation sequencing have mainly focused on the plastid genome, few involved the nuclear genome. By analyzing whole-genome datasets from the Poaceae, one of them identified 74 putative nuclear single copy orthologous genes for phylogenetic studies of temperate bamboos^22^, but this method is labor-intensive. With the development of high-throughput sequencing technologies, reduced-representation methods have revolutionized the fields of phylogeography, population genomics, and phylogenomics by providing high-resolution genomic data for non-model organisms at a reasonable cost^23–25^, such as restriction site-associated DNA sequencing (RAD-seq)^26^. By reducing genomic representation, RAD-seq can identify tens of thousands of single nucleotide polymorphism (SNP) markers, and address the issue of phylogenetic reconstruction with unprecedented power and precision, even with limited, or no reference genome^27–32^. Many empirical studies have employed this method on plants and animals to reconstruct their phylogenetic relationships and demonstrated its power on phylogenetic resolution in non-model organisms^30, 32–35^. Therefore, RAD-seq provides an opportunity to solve the contentious relationships of Arundinarieae from nuclear evolutionary trajectory.

Mapping and grouping are two SNP-calling systems for obtaining large numbers of SNPs from RAD sequencing data. In mapping, RAD sequencing reads are aligned to a reference genome and genotyped using standard tools, such as BWA^36^ and Stampy^37^, and the output alignments are supported by several generic SNP callers such as Genome Analysis Tool Kit^38^ (GATK) and SAMtools^39^. In grouping, RAD sequencing reads are used *de novo*, generating large marker sets where no reference genome is available. Several tools have been developed to produce RAD marker sets *de novo*, including Stacks^40^ and RADtools^41^. Pan *et al*.^42^ tested and compared SNP calling using the UNEAK, Stacks and bowtie2-GATK pipelines for genotyping-by-sequencing (GBS) data in nine individuals of the three pine species, and found that both Stacks and bowtie2-GATK were more efficient than UNEAK for SNP calling. However, to date, there has been no comparison of the performance of mapping and grouping in terms of the variants obtained and downstream phylogenetic analysis of RAD sequencing data.

In a pilot study, we elucidated the phylogenetic relationship between two closely related species in temperate bamboos using RAD sequencing^43^. However, the utility of RAD-seq in building Arundinarieae phylogeny when more taxa sampled remains elusive. The *Phyllostachys* clade (clade V) is the largest clade in Arundinarieae, with ca. 16 genera and more than 330 species which comprises about 50% of the genera and more than 70% of the species of the tribe^15, 44^. The clade is remarkable for combining high morphological diversity with low plastid DNA variability. Therefore we adopt broad taxon sampling with an emphasis on *Phyllostachys* clade and related clades to elucidate their phylogenetic relationships, which would act as a valuable starting point for reconstructing a comprehensive phylogenetic framework for the whole tribe. The primary goals of this investigation were (1) to test the utility of RAD data in providing a high-resolution estimate of the phylogenetic relationships among temperate bamboos, where a broad sample was examined; and (2) to evaluate and compare mapping and grouping systems for SNP calling and phylogeny inference based on RAD sequencing data.

## Results

### RAD sequencing

We obtained an average of 11.0 million paired-end reads of 82–86 bp per sample and approximately 615 million reads in all after barcode trimming, cleaning and quality checking. Details of the sequencing output are provided in Supplementary Table S1.

### Data matrices from mapping system

Using BWA, we were able to map between 6.58% (*Guadua angustifolia*) and 99.12% (*Phyllostachys edulis*) (mean = 57.55%) of the RAD tags to the genomic scaffold sequences. The reference-based GATK HaplotypeCaller identified 6,602,640 raw variants. Filtering for a coverage of 10 to 500 resulted in 5,934,688 variants being retained. Only 1390 variable sites were obtained when we set the strictest limit (0) for ‘number of no-called samples’ (matrix s56, requiring all the 56 samples to have data at each locus). In order to maximize the number of loci that could be analyzed we applied multiple threshold values for the ‘number of no-called samples’ (NCC) = 1, 5, 10 in further analyses. For example, matrix s55 (NCC = 1, requiring 55 of the 56 samples to have data at each locus) contains only 1.34% missing data, but only contains 5759 variable sites (Table 1). The characteristics of the data matrices produced in this method are outlined in Table 1. The four data matrices ranged from 2357 bp (1390 variable sites and 489 informative sites in s56) to 400,796 bp (272,254 variable sites and 97,104 informative sites in s45), and the proportion of missing data in the matrices ranged from 0 (s56) to 13.08% (s45) (Table 1).

**Table 1:** Summary of data matrices produced through BWA-GATK pipeline.

Matrix^*^	missing data	base pairs	SNPs	informative SNPs
s56	0	2357	1390	489
**s55**	1.34%	9416	5759	2076
**s50**	7.64%	147524	97588	35056
**s45**	13.08%	400796	272254	97104

^*^Minimum number of samples needed to retain a locus (total number of samples is 56). The data matrices used for phylogenetic analyses are shown in bold.

### Data matrices from grouping system

As Stacks only infers loci from forward reads, reverse reads were not included for genotyping. Setting the minimum coverage depth to 5 in *ustacks* produced between 39,291 (*Indosasa sinica*) and 496,805 (*Dendrocalamus latiflorus*) putative loci, with an average of 186,392 loci (Supplementary Table S2). Increasing the minimum stack depth to 10 produced between 6003 (*I*. *sinica*) and 308,783 (*P*. *edulis*) putative loci, with an average 57,507 (Supplementary Table S2). With the options -m 5 and 10, a total of 4,715,488 and 972,878 putative loci (cut sites), respectively, were produced among the 56 samples.

In this method, we generated a total of 14 SNP matrices (-m 5, 10 and -p 10, 15, 20, 25, 35, 45, 55) outlined in Table 2, which ranged in total sequence length from 0 bp to 362,823 bp (m = 5) and 9 bp to 42,788 bp (m = 10). Applying higher values of minimum stack depth tended to decrease the overall concatenated matrix length. The proportion of missing data in these matrices ranged from 0 to 72.64% at m = 5 and from 1.79% to 73.87% at m = 10 (Table 2). The coverage values are high, indicating that the sequencing is unlikely to be the main contributor to the high levels of missing data that we observed. One of the outgroups used in our study (*G*. *angustifolia*) consistently had the highest proportion of missing data. This may be caused by high levels of molecular divergence between *G*. *angustifolia* and the rest of the species analyzed.

### Phylogenetic inference

**Figure 1:**
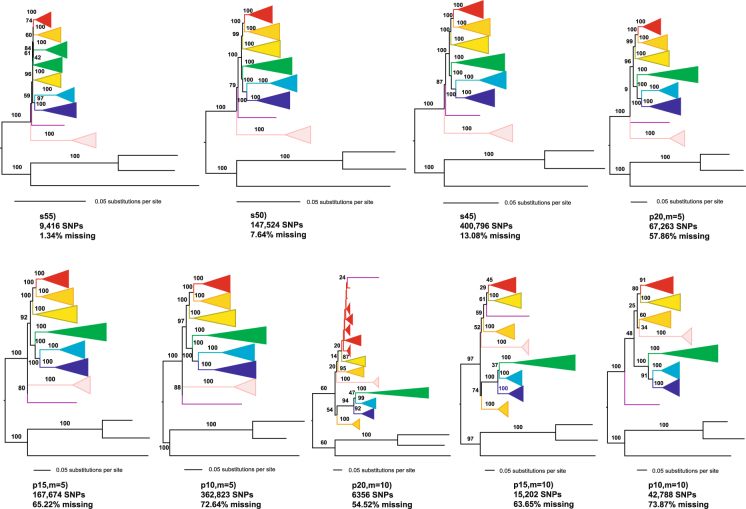
Tree resolution increases as data are added to the phylogenetic analysis. The different colors represent eight major clades (see Fig. 2 for key). Values on branches are bootstrap support from 200 bootstrap replicates using RAxML’s rapid bootstrap algorithm. Topologies shown are the best tree from a full ML search. Bootstrap support values within major clades are not shown.

**Figure 2:**
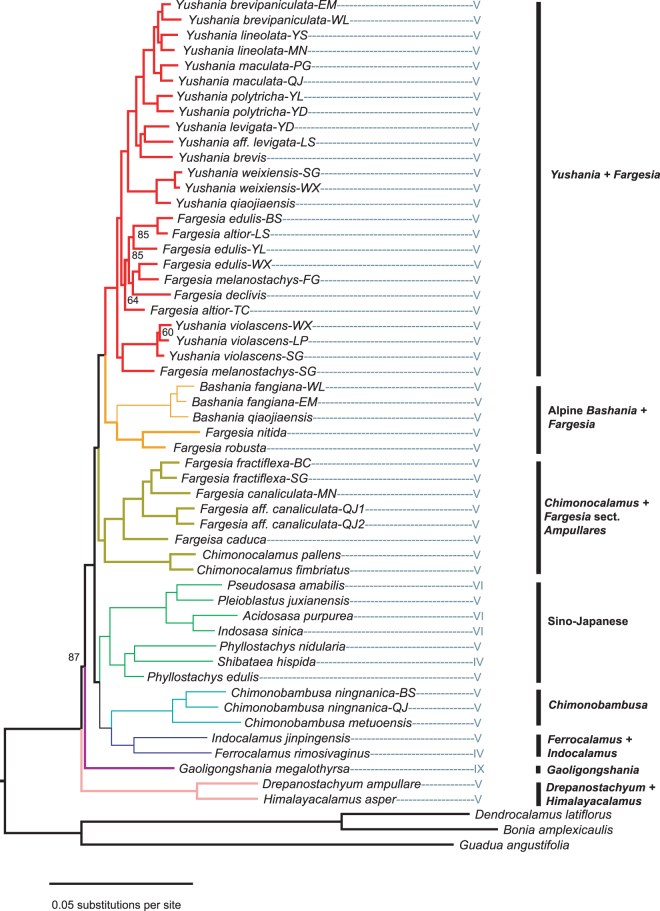
Phylogeny of the temperate bamboos based on an ML analysis (matrix s45: 400,796 base pairs, and 272,254 variable sites). Nodes with posterior probalilities ≥95 are unlabeled. Rhizome type is indicated by branch thickness: thick lines = pachymorph rhizomes and thin lines = leptomorph rhizomes.

We estimated phylogenetic trees (Fig. 1) for the temperate bamboos with nine representative data matrices selected from total eighteen matrices generated from both genotyping systems, which contained varying levels of missing data (Tables 1 and 2). Among the trees, eight monophyletic lineages can be discerned within the tribe with strong support. The eight lineages were designated: *Drepanostachyum* + *Himalayacalamus*, *Gaoligongshania*, *Ferrocalamus *+* Indocalamus*, *Chimonobambusa*, Sino-Japanese lineage, *Chimonocalamus *+* Fargesia* sect. *Ampullares*, alpine *Bashania *+* Fargesia*, and *Yushania *+* Fargesia* (detailed in Fig. 2).

**Table 2:** Summary of data matrices produced through Stacks pipeline.

Matrix^*^		missing data	SNPs	informative SNPs	total loci	consensus loci	polymorphic loci
m = 5	s55	0	0	0	2	2	0
	s45	13.59%	97	46	119	68	51
	s35	29.29%	911	241	456	149	307
	s25	50.14%	20855	6485	4651	319	4332
	**s20**	57.86%	67263	21357	13889	538	13351
	**s15**	65.22%	167674	53927	33678	1082	32596
	**s10**	72.64%	362823	119331	74448	2856	71592
m = 10	s55	1.79%	9	6	10	6	4
	s45	10.78%	208	90	245	132	113
	s35	24.31%	537	182	433	192	241
	s25	44.50%	2589	777	1365	381	984
	**s20**	54.52%	6356	1957	2780	639	2141
	**s15**	63.65%	15202	4942	5665	1069	4596
	**s10**	73.87%	42788	14194	14097	2198	11899

^*^p = minimum numbers of populations required to process a locus. The data matrices used for phylogenetic analyses are shown in bold.

For the reference-based genotyping data sets, phylogenetic analysis using the smallest data set (s55; 9416 bp; Table 1) produced topologies with low bootstrap support, and did not form eight major clades (Fig. 1 and Table 3). This pattern changed dramatically as more data were added to the analysis (Fig. 1). Analyses with data matrices of at least 147,524 bp resolved the eight principal lineages as monophyly with a bootstrap support of 100%, with one exception. The *Gaoligongshania* lineage had a bootstrap support of 79% and 87% in the s50 and s45 data matrix, respectively (Fig. 1 and Table 3).

**Table 3:** Bootstrap support for relationships within Bambusoideae.

Clade	Genus	s55	s50	s45	p20, m = 5	p15, m = 5	p10, m = 5	p20, m = 10	p15, m = 10	p10, m = 10
*Drepanostachyum* + *Himalayacalamus*	*Drepanostachyum*, *Himalayacalamus*	100/97	100/98	100/99	100/98	100/100	100/94	100/98	100/100	100/96
*Gaoligongshania*	*Gaoligongshania*	59/59	79/94	87/97	9/84	80/86	88/75	24/25	29/−	48−
*Ferrocalamus* + *Indocalamus*	*Ferrocalamus*, *Indocalamus*	100/97	100/97	100/95	100/−	100/−	100/50	92/−	100/−	100/−
*Chimonobambusa*	*Chimonobambusa*	100/98	100/96	100/95	100/100	100/100	100/100	99/61	100/61	100/74
Sino–Japanese	*Acidosasa*, *Indosasa*, *Phyllostachys*, *Pleioblastus*, *Pseudosasa*, *Shibataea*	−/49	100/92	100/90	100/100	100/100	100/98	100/−	100/15	100/29
*Chimonocalamus* + *Fargesia* sect. *Ampullares*	*Chimonocalamus*, *Fargesia*	100/73	100/88	100/91	100/77	100/98	100/83	87/44	100/−	100/−
Alpine *Bashania* + *Fargesia*	*Bashania*, *Fargesia*	100/93	100/96	100/99	100/67	100/92	100/77	−/−	−/−	60/−
*Yushania* + *Fargesia*	*Fargesia*,*Yushania*	100/100	100/99	100/100	100/100	100/100	100/100	−/−	45/−	91/−

Bipartitions that were absent are represented by a “−” Results are shown for two approaches: concatenated/species tree.

**Figure 3:**
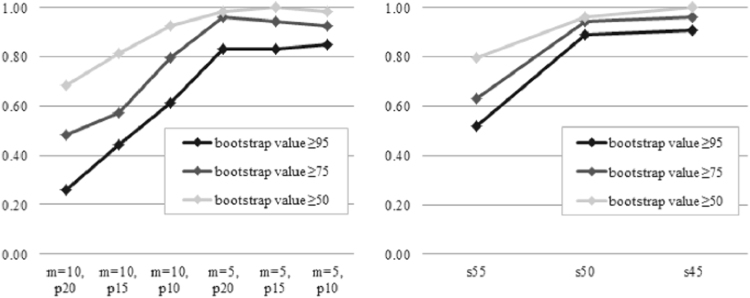
The resolution of phylogenetic trees increases as a function of data matrix size. The colors indicate the proportion of branches with a bootstrap support above a given threshold.

Similar patterns were observed in the *de novo* assembly genotyping data sets. As the data matrix increased in size, the resolution of these clades also increased, along with the bootstrap support of the internal branches of the trees (Figs 1 and 3). Decreasing the minimum stack depth dramatically increased the size of the data matrix. Phylogenetic analysis using the smallest data set (m = 10, p = 20; 6356 SNPs; Table 2) produced topologies with very low bootstrap support, and only five of the eight lineages described above formed clades, with bootstrap supports of 87–100% (Fig. 1). The topology changed as more data were added. In the analysis of data matrix p15 (m = 5), the eight principal lineages were monophyletic, with a bootstrap support of 100%, with the exception of the *Gaoligongshania* lineage, which had a bootstrap support of 80%. The relatively low bootstrap support for the monophyly of this clade was not found in other analyses. For data matrices p15 (m = 5) and p10 (m = 5), we found only minimal differences in the supporting values at a few of the more derived branches. We noted there was a difference in topology in the derived *Gaoligongshania* lineage and *Drepanostachyum* + *Himalayacalamus* lineage between the largest data matrices p10 (m = 5, Stacks pipeline) and s45 (BWA + GATK pipeline) (Fig. 1).

We compared the support values for all shared bipartitions between data matrices with different proportions of missing data (Fig. 3). The total number of well-supported branches on the tree generally increased as a negative exponential function of the number of base pairs in the data matrix, with two exceptions (Fig. 3). In analyses of p15 (m = 5), the number of branches with 75% or greater support decreased slightly, while in p10 (m = 5) the number of branches with 50% or greater support slightly decreased. Notably, the tree length estimates obtained using the *de novo* assembly genotyping data matrices were substantially higher in comparison to those from the reference-based genotyping data matrices (Table 4).

**Table 4:** Data characteristics with bootstrap value and tree length for each data matrix.

Matrix	Mean ML bootstrap	tree length
s55	77.52	0.78
s50	96.15	1.06
s45	97.80	1.23
p20, m = 5	95.33	3.26
p15, m = 5	96.26	3.89
p10, m = 5	95.76	4.73
p20, m = 10	66.06	4.60
p15, m = 10	76.33	4.97
p10, m = 10	87.56	5.32

### Phylogenomics of Temperate bamboos

Summaries of the phylogenetic trees from analyses of the nine data matrices using RAxML are provided in the Table 4. The ML phylogenetic analysis of the largest data matrix (s45) is shown in Fig. 2. Neither the *Shibataea* (IV) nor *Arundinaria* (VI) clades formed monophyletic groups, nor did *Phyllostachys* (V). The current data provided a test of the monophyly of six genera (i.e., those for which at least two species were sampled). Of these, only three were strongly supported as monophyly (*Chimonocalamus*, *Chimonobambusa*, and Alpine *Bashania*), while three appear to be paraphyletic or polyphyletic (*Fargesia*, *Yushania*, and *Phyllostachys*), which corroborates earlier studies^16, 18, 44^. At the species level, *Chimonobambusa ningnanica*, *Bashania fangiana*, and all of the species within *Yushania* were resolved as monophyletic. Intrageneric relationships in *Fargesia* were unresolved, and none of the sampled species appeared to be monophyletic.

**Figure 4:**
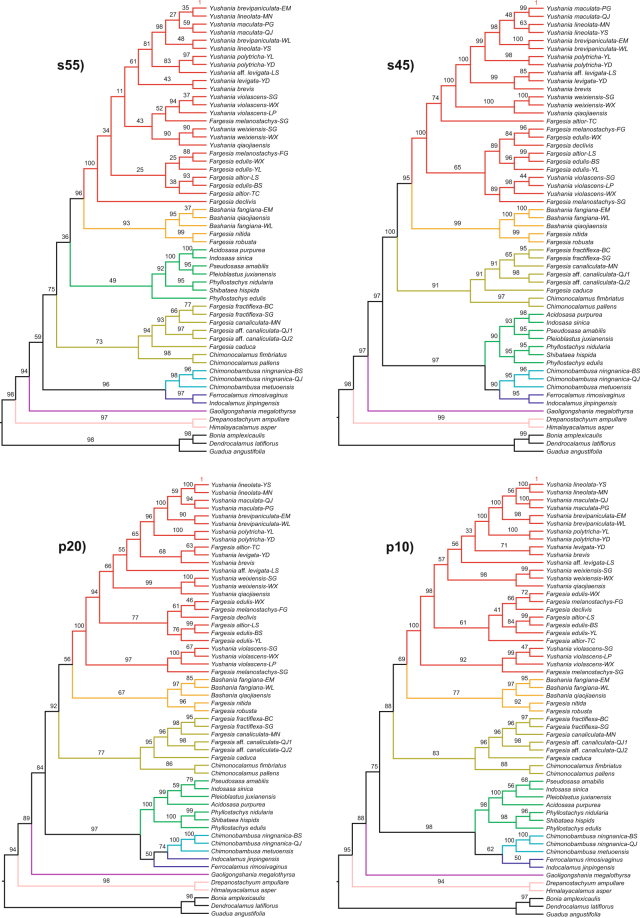
Species trees from temperate bamboos estimated using SVDquartets for data matrices s55, s45, p20 (m = 5), p10 (m = 5). The different colors represent eight major clades (see Fig. 2 for key). Bootstrap values (from 100 replicates) are shown on nodes.

The species trees estimated using SVDquartets were largely similar to each other with four different data matrices, with a few notable exceptions (Fig. 4). First, the phylogenetic placement of the Sino-Japanese and *Chimonocalamus* + *Fargesia* sect. *Ampullares* lineages were not consistent. Second, the genera *Ferrocalamus* and *Indocalamus* did not form a clade when the smallest data matrix was used, but the two genera formed a clade in all other data matrices. Finally, the relationships within the *Yushania* + *Fargesia* and Sino-Japanese lineages varied across different data matrices. We noted that the phylogenetic placement of *Drepanostachyum* + *Himalayacalamus* and *Gaoligongshania* lineages were consistent across all data matrices. The species tree estimated using the largest matrix was largely similar to the expected ML phylogeny, except for some short internal branches in the *Yushania* + *Fargesia* lineage.

## Discussion

The temperate bamboos have long been considered a complex and taxonomically difficult group. Previous phylogenetic studies mainly based on plastid DNA and divided Arundinarieae into twelve clades, but few was known about the nuclear evolutionary history due to unusable of most single or low copy genes in bamboos and the low molecular variation^14, 19, 45^. In this study, we sampled 39 species and the results of our phylogenetic analyses with RAD-seq data provided new insights into the phylogenetic relationships of Arundinarieae, mainly involving the *Phyllostachys* clade and its closely related clades. Eight major lineages were identified in Arundinarieae with high support, two of which (*Drepanostachyum* + *Himalayacalamus*, and *Chimonobambusa*) are consistent with previous results based on nuclear genes^16, 18^, while others were recovered here for the first time. Such clear relationships among these clades of Arundinarieae demonstrated that RAD-seq is a powerful tool for solving this intricate group by providing tens of thousands of SNPs scatter in the whole genome.

Compared with plastid phylogeny, many inconsistencies were revealed in our study. The *Phyllostachys*, *Shibataea*, and *Arundinaria* clades defined from plastid phylogeny were not supported as monophyly anymore. Instead, they were separated into several different parts in the nuclear tree. Incomplete lineage sorting and/or hybridization (introgression) could be the cause for gene tree conflicts in temperate bamboos, as summarized by Zhang *et al*.^18^. In the plastid phylogeny, division of lineages was incompatible with traditional classification based on morphology, and the genera delimited based on these characters were not monophyletic, which has confused taxonomists for a long time. However, our nuclear phylogeny agrees with the morphology-based taxonomy to some extent, which has also been observed in other organisms^46, 47^. Two clades with leptomorph rhizomes were strongly supported as monophyletic (Fig. 2). One included the Sino-Japanese, *Ferrocalamus* + *Indocalamus* and *Chimonobambusa*, among them *Chimonobambusa* has leptomorph rhizomes and highly reduced culm-sheath blades. This genus was resolved as monophyletic group which is congruent with morphologic delimitation. The other was the alpine *Bashania*, three species from high mountains formed a strongly supported group. This result supported the combination of new genus *Sarocalamus* described by Stapleton *et al*.^48^ with a distribution from the eastern Himalayas to the Sichuan Province of China.

*Fargesia* and *Yushania* are two genera of alpine bamboos^14^, mainly distributed in the Hengduan Mt. They all produced pachymorph rhizomes and semelauctant synflorescences, and the length of culm-necks and the type of synflorescence are important characters for genera delimitation. However, such delimitation was confused because of the variability of the length of culm necks and the open degree of spathe subtending observed in the field. Whether to recognize *Fargesia* and *Yushania* as genera was still in debate. In our phylogeny, *Yushania violascens* was nested with *Fargesia* species with high bootstrap support. These taxa have intermediate morphology and hybridization may be a likely explanation for these taxonomic uncertainties in this group. Thus, *Yushania* might be a good genus for traditional taxonomists. With respect to *Fargesia*, it was separated into three parts. The *Fargesia* section *Ampullares*, as defined by Yi^49^ was resolved as monophyletic here and supported by morphological characters. All individuals in this section possess semi-orbicular (broadly ovoid) culm buds and numerous and congested branches. While *Fargesia fractiflexa* was transferred into *Drepanostachyum* as *Drepanostachyum fractiflexum* because of many branches with underdeveloped secondary branches in Flora of China^6^. According to the nuclear phylogeny, *F*. *fractiflexa* had a close relationship with *Fargesia*, and it was more reasonable to transfer *D*. *fractiflexum* back into *Fargesia*. *Fargesia caduca* was found in the South Yunnan of China and placed in section *Fargesia* because of bud characters^49^. As we observed in the field, *F*. *caduca* indeed exhibited the complete range of variation from ovoid to lanceolate buds, and might be an intermediate species.

Additionally, our work highlights the fact that different conclusions can be reached in phylogenetic inference using data sets generated from two different pipelines. Using data generated from 56 samples representing 36 species of temperate bamboo, we observed significant inconsistencies between the Stacks and BWA-GATK pipelines. These inconsistencies in the SNP datasets led to strong disagreement in downstream phylogenetic inferences. Both Stacks and BWA-GATK were efficient at SNP calling, identifying 2,255,239 and 5,934,688 SNPs in the samples, respectively. For the same level of missing data, the BWA-GATK pipeline produced much more SNPs in comparison with Stacks. One possible reason is that BWA-GATK used both forward and reverse reads, while Stacks only used forward ones. Moreover, compared to the BWA-GATK pipeline, Stacks suffered from a higher rate of missing data, with up to 100% of the SNPs recovered by Stacks having missing genotypes among the samples. The high proportion of missing genotypes of Stacks was linked to several characteristics of the pipeline, including non-gapped alignment, the requirement for a certain coverage depth per locus per sample, and an arbitrary and low sequence divergence threshold between alleles of a locus^40^. However, BWA-GATK pipeline allows for gaps and has a higher sequence divergence threshold in alignments^50^, which significantly reduces the proportion of SNPs with missing genotypes. A strategy incorporating both Stacks and BWA-GATK can take advantage of the unique properties of the two methods to perform more efficiently than either tool alone, especially in SNP calling for samples with high levels of genetic divergence.

RAD-seq was suggested as a powerful tool for interspecific phylogeny reconstruction by several simulated and empirical studies^30, 33, 51^. But calling SNPs without a reference genome is difficult and requires careful analysis to develop an optimal strategy. As seen here, the threshold of coverage depth required to create a stack had strong influence on the size of the final data matrix and inferred phylogenetic relationships (Table 2 and Fig. 1). We found conflicting topologies and variable levels of bootstrap support when changing the minimum stack depth and the minimum number of samples needed to retain a locus in the final alignment. Phylogenetic analyses using data matrices p20 and p15 (m = 10) produced different topologies with very low bootstrap support. When the minimum stack depth was reduced to five (m = 5), the phylogenetic relationships and bootstrap values were more stable across the different proportions of missing data (Fig. 1). This suggests that setting -m too high could miss too many information to cause incorrect inferences of sample differentiation^52, 53^. The possible cause for these missing information is that many reads with errors exist in duplicate and are labeled as stacks in the initial hashing stage of the algorithm when the minimum stack depth is five, but they would be forced into other loci when increasing the minimum stack depth to ten^54^. In this situation, stacks that truly have a depth of only five get lost, but including a larger sample of individuals with greater accuracy and precision for population parameters which would compensate for low coverage at individual loci in phylogenetic analyses^27, 52^. The results presented here demonstrate that setting the minimum stack depth to a higher value (e.g., ten) would not be beneficial for increasing precision and accuracy in phylogenetic analysis, but a lower one did.

Accompanying with the change of parameter setting, the number of loci containing missing data in the final sequence matrices altered and would influence the resolution and supports of the temperate bamboo phylogenies greatly. RAD loci usually contain large amounts of missing data, and this problem is more pervasive for distantly related species due to allelic dropout^55^. Assembling reads from next-generation sequencing data, either *de novo* or by mapping to a reference genome, relies on sequence similarity^40^. Hence, alleles with greater divergence among individuals (or relative to the reference genome) may be excluded, especially for more distantly related taxa. Meanwhile, the amount of missing data in the final data matrix is controlled by parameters which set by users, and applying a lower tolerance for missing data comes at the cost of retaining far fewer loci^32^. Thus, the number of SNPs acquired is positively correlated with missing data, matrices containing minimal missing data and relatively few SNPs produced topologies with extremely low bootstrap support (Fig. 1). In addition, as the tolerance for missing data becomes more stringent, the mutational spectrum represented in the sampled loci was truncated, leading to the disproportionately exclude of the loci with highest mutation rates^56^. However, concatenating more RAD loci generally obtained higher bipartition supports though with large amount of missing data, a phenomenon observed in both empirical^24, 32, 33, 57^ and simulated^35, 51, 56^ phylogenetic studies. Data processing directly impacts the size of the data matrix and therefore the phylogenetic reconstruction and generally larger data sets even if with high proportion of missing data can lead to more accurate inferences.

In conclusion, our study is one of the first to explore the utility of RAD sequencing technology in estimating phylogenetic relationships among Arundinarieae genera. With an emphasis on the *Phyllostachys* clade, we produce phylogenetic trees with unprecedented resolution for the temperate bamboos and demonstrate that RAD sequencing appears a promising tool for resolving difficult phylogenetic relationships for intractable plant groups. In future, with more broad taxa sampling the RAD sequencing could be used to reconstruct a comprehensive phylogenetic framework for the temperate bamboos. Our work also highlights the sensitivity of phylogenetic inferences to the parameter settings used during SNP genotyping. Careful attention to the analysis pipeline is vital for SNP calling and downstream phylogenetic analyses.

## Materials and Methods

### Taxon Sampling

Temperate bamboos are a diverse clade containing 19–31 genera and approximately 546﻿ species. A total of 56 populations representing 39 species in 19 genera were sampled for this study (Supplementary Table S1). Thirty species were from clade V, two from clade IV, three from clade VI, and one from clade IX. Given the monophyly of the temperate bamboos, three species of tropical woody bamboos (Bambuseae), *Bonia amplexicaulis*, *D*. *latiflorus*, and *G*. *angustifolia*, were chosen as outgroup taxa. The tropical and temperate bamboos are hexaploids (2n = 72), t﻿etraploids (2n=46) and tetraploids (2n = 48), respectively. All species were collected from natural populations, with two exceptions: *Himalayacalamus asper*, which was collected from Tradewinds Bamboo Nursery (http://www.bamboodirect.com/); and *G*. *angustifolia*, which was collected from the Xishuangbanna Tropical Botanical Garden of the Chinese Academy of Sciences. Vouchers of all collections were deposited in the herbarium of the Kunming Institute of Botany. Plant material was dried in silica gel to minimize DNA degradation. Four to six individuals per populations were sampled, with the exception of *H*. *asper*, *B*. *amplexicaulis*, *D*. *latiflorus*, *G*. *angustifolia*, and *P*. *edulis*, where only one individual was available.

### RAD tag library construction and sequencing

Total genomic DNA was extracted from silica gel-dried leaf material using a modified CTAB procedure^58^. Genomic DNA was pooled from one to six individuals in each population to form population samples as described in Emerson *et al*.^24^ and Hohenlohe *et al*.^59^. In brief, sequencing adaptors and populations barcodes were ligated to *EcoR*I-digested total genomic DNA, and the resulting fragments were sequenced from the restriction sites. RAD libraries were prepared and sequenced according to Miller *et al*.^60^ and Baird *et al*.^26^. All libraries were sequenced on an Illumina HiSeq. 2000 platform, with paired-end sequencing and a read length of 91 bp. RAD sequences were pre-processed through two quality filtering steps to exclude reads which contained contaminated adapter sequences or had more than 50% of base calls with a low quality score (Q ≤ 5). Reads were then de-multiplexed by sorting them into the 56 samples according to their barcodes. Construction of RAD libraries, Illumina HiSeq. 2000 sequencing, raw data cleaning and quality control were performed at BGI Shenzhen, China.

### Mapping system: reference-based genotyping approach

The genome of one species of temperate bamboo, *P*. *edulis* (*Phyllostachys heterocycla*), has recently been published^61^. We used the available *P*. *edulis* whole genome (http://www.ncgr.ac.cn/bamboo) as a reference for mapping and to identify SNPs. Sequencing reads (both paired-end 82–86 bp fragments) were aligned to the reference using BWA^36^ (v0.7.5). Default parameters were used, allowing up to four mismatches and one gap when aligning reads to the genome. Alignments were converted from sequence alignment map (SAM) format to sorted, indexed binary alignment map (BAM) files^62^ (SAMtools v0.1.18). The Picard tool (v1.103; http://broadinstitute.github.io/picard) was used to remove duplicate reads. Genotypes were called for all samples individually with HaplotypeCaller from the Genome Analysis Tool Kit (GATK)^38^ (v3.3.0) using the default settings. The variant calls were filtered using a minimum PHRED quality threshold of 20 and a minimum and a maximum variant coverage of 10 and 500 reads, respectively. To examine the balance between obtaining a large number of SNPs and minimizing missing data, we exported four data sets that contained varying levels of missing data by adjusting the “number of no-called samples” parameter (NCC = 0, 1, 5, 10).

### Grouping system: *de novo* assembly genotyping approach

Reads with uncalled nucleotides were discarded using the *process_radtags* script from the Stacks pipeline^40^ (v1.08). Within each population, *de novo* assembly was conducted using *ustacks*. We set the maximum number of mismatches allowed between stacks (-M) to 3. The *cstacks* program was used to build a catalog from all populations with three mismatches among loci were allowed (n = 3), and then *sstacks* was used to search against the catalog produced by *cstacks*. Genotypes were called for all populations using the Stacks *populations* program. To assess the influence of coverage depth and the amount of missing data on the outcome of the data matrices and phylogenetic inferences, we used different combinations of parameters, including (1) two different minimum depths of coverage required to form a stack (-m 5, 10); and (2) seven different minimum numbers of populations required to process a locus (-p 10, 15, 20, 25, 35, 45, 55).

### Maximum likelihood phylogenetic analyses

We conducted phylogenetic analyses with concatenated SNP datasets using the maximum likelihood (ML) method. The species *B*. *amplexicaulis*, *D*. *latiflorus*, and *G*. *angustifolia* were used as outgroups. Maximum likelihood analyses were implemented using RAxML^63^ (v7.2.8). Non-parametric bootstrapping was implemented using the fast bootstrap algorithm of RAxML with 200 replicates. The data matrices were analyzed using the GTR + Γ model of nucleotide evolution (GTRGAMMA), as recommended by the authors of the program. Trees were visualized and edited in FigTree (version 1.3.1; http://tree.bio.ed.ac.uk/software/figtree/). We extracted measures of total tree length from the results files (RAxML info files), as well as branch lengths and bootstrap values from the RAxML bipartition tree files.

### Species tree estimation

We used the program SVDquartets^64^ (v1.0) to estimate the coalescent-based species tree using the RAD loci. An advantage of this approach for analyses of RAD data is that it seems to be able to handle large amounts of missing data^35^. We applied SVDquartets to six data matrices, p10 (m = 5), p15 (m = 5), p20 (m = 5), s45, s50, and s55. For each data matrix, we randomly sampled 100,000 quartets from the 56 populations. The quartet program Quartet MaxCut^65^ (v2.1.0) was used to infer the species tree from the sampled quartets. We used nonparametric bootstrapping with 100 replicates to measure uncertainty in bipartitions. The bootstrap values were mapped to the species tree estimated from the original data matrix using SumTrees^66^ (v3.3.1).

### Data accessibility

The raw ﻿data of four samples (AEM, AWL, YEM, YWL) are available on Dryad (doi:10.2061/dryad.lmj31), and the rest data files are available from the National Center for Biotechnology Information (NCBI) Sequence-Read Archive (SRA) database with accession numbers﻿ SRR6001006-SRR6001057.

## Electronic supplementary material

Supplementary Information
