# Supplementary material for: Genome-wide RAD sequencing data provide unprecedented resolution of the phylogeny of temperate bamboos (Poaceae: Bambusoideae)
Source: Sci Rep. 2017 Sep 14;7:11546. doi: 10.1038/s41598-017-11367-x (PMC5599554; doi:10.1038/s41598-017-11367-x)
Supplement: Supplementary file 1 — Supplementary Information [file 41598_2017_11367_MOESM1_ESM.pdf]

Genome-wide RAD sequencing data provide unprecedented resolution of the phylogeny of temperate bamboos (Poaceae: Bambusoideae)

Xueqin Wang[1,2*], Xiaying Ye[1,3*], Lei Zhao[1,3], Dezhu Li[1,4], Zhenhua Guo[1] & Huifu Zhang[5]

[1]Plant Germplasm and Genomics Center, Germplasm Bank of Wild Species, Kunming Institute of Botany, Chinese Academy of Sciences, Kunming 650201, China.

[2]College of Life Science and Agronomy, Zhoukou Normal University, Zhoukou 466001, China.

[3]Kunming College of Life Sciences, University of Chinese Academy of Sciences, Kunming 650201, China.

[4]Key Laboratory of Biodiversity and Biogeography, Kunming Institute of Botany, Chinese Academy of Sciences, Kunming 650201, China.

[5]Key Laboratory of Economic Plants and Biotechnology, Yunnan Key Laboratory for Wild Plant Resources, Kunming Institute of Botany, Chinese Academy of Sciences, Kunming 650201, China.

[*] These authors contributed equally to this work.

Correspondence and requests for materials should be addressed to D.Z. L. (email: dzl@mail.kib.ac.cn) or Z.H. G. (email: guozhenhua@mail.kib.ac.cn)

Table S1 Plant materials used.

Samples	Voucher	Clade	Location	Latitude	Longitude	Altidude (m)	Reads (M)
temperate bamboos							
Ferrocalamus rimosivaginus	wxq126	IV	Jinping, Yunnan	N22 °53'34"	E103 °24'55"	719	6.87
Shibataea hispida	SH	IV	Xiuning, An'hui	N29 °42'03"	E117 °47'16"	467	5.52
Bashania fangiana-EM	AEM	V	E'mei, Sichuan	N29 °31'52"	E103 °19'28"	2826	27.15
Bashania fangiana-WL	AWL	V	Wolong, Sichuan	N31 °02'36"	E103 °09'50"	2696	27.08
Bashania qiaojiaensis	wxq149	V	Qiaojia, Yunnan	N27 °01'52"	E103 °00'47"	2760	9.89
Chimonobambusa metuoensis	123480	V	Motuo, Xizang	N29 °39'11"	E95 °29'31"	1960	13.57
Chimonobambusa ningnanica-BS	wxq091	V	Baoshan, Yunnan	N24 °50'08"	E98 °45'50"	2147	10.22
Chimonobambusa ningnanica-QJ	wxq100	V	Qiaojia, Yunnan	N27 °05'45"	E103 °02'44"	2200	4.73
Chimonocalamus fimbriatus	wxq128	V	Jinping, Yunnan	N22 °51'07"	E103 °25'03"	1322	5.44
Chimonocalamus pallens	wxq130	V	Jinping, Yunnan	N22 °51'26"	E103 °13'15"	1916	8.49
Drepanostachyum ampullare	123651	V	Zhangmu, Xizang	N27 °58'44"	E85 °58'10"	2219	6.35
Fargesia altior-LS	wxq079	V	Lushi, Yunnan	N25 °59'06"	E98 °49'12"	2500	11.32

Fargesia altior-TC	wxq084	V	Tengchong, Yunnan	N25°39'23"	E98°26'29"	2385	8.06
Fargesia caduca	wxq139	V	Jinggu, Yunnan	N23°39'44"	E100°42'31"	1873	4.85
Fargesia canaliculata-MN	Z11052	V	Mianning, Sichuan	N28°13'48"	E101°52'04"	1483	6.32
Fargesia aff. *canaliculata-QJ1*	wxq098	V	Qiaojia, Yunnan	N26°51'59"	E103°00'39"	2113	7.61
Fargesia aff. *canaliculata-QJ2*	wxq106	V	Qiaojia, Yunnan	N26°51'44"	E103°00'42"	2192	5.58
Fargesia declivis	Z11026	V	Gongshan, Yunnan	N27°44'59"	E98°20'48"	1387	5.43
Fargesia edulis-BS	wxq093	V	Baoshan, Yunnan	N25°17'47"	E98°46'46"	2200	7.30
Fargesia edulis-WX	wxq059	V	Weixi, Yunnan	N27°21'27"	E99°20'50"	2820	11.05
Fargesia edulis-YL	wxq070	V	Yunlong, Yunnan	N25°45'34"	E99°05'12"	2639	7.82
Fargesia fractiflexa-BC	wxq050	V	Binchuan, Yunnan	N25°55'30"	E100°25'14"	1856	5.30
Fargesia fractiflexa-SG	wxq054	V	Shanggeri-la, Yunnan	N27°10'11"	E100°03'41"	1825	15.63
Fargesia melanostachys-FG	Z11014	V	Fugong, Yunnan	N27°10'36"	E98°45'02"	3254	6.30
Fargesia melanostachys-SG	wxq056	V	Shanggeri-la, Yunnan	N27°25'24"	E99°50'46"	3241	10.75
Fargesia nitida	FN	V	Wolong, Sichuan	N30°52'06"	E102°58'46"	2777	15.89
Fargesia robusta	wxq002	V	Wolong, Sichuan	N31°01'52"	E103°10'41"	2050	5.03
Himalayacalamus asper	HA	V	India				5.19

Indocalamus jinpingensis	wxq129	V	Jinping, Yunnan	N22°51'14"	E103°24'32"	1450	5.68
Phyllostachys edulis	PE	V	Tianmuxhan, Zhengjiang	N30°19'13"	E119°36'55"	480	15.17
Phyllostachys nidularia	wxq016	V	Dujiangyan, Sichuan	N30°57'08"	E103°24'39"	1224	9.76
Pleioblastus juxianensis	PJ	V	Yuexi, An'hui	N30°50'21"	E116°16'39"	666	5.50
Yushania brevipaniculata-EM	YEM	V	E'mei, Sichuan	N29°31'59"	E103°19'57"	2600	27.09
Yushania brevipaniculata-WL	YWL	V	Wolong, Sichuan	N31°01'34"	E103°11'13"	2476	27.23
Yushania brevis	wxq131	V	Luchun, Yunnan	N22°59'16"	E102°27'13"	1960	7.65
Yushania levigata-YD	wxq096	V	Yongde, Yunnan	N24°10'29"	E99°38'17"	2644	12.44
Yushania aff. levigata-LS	wxq076	V	Lushui, Yunnan	N26°00'22"	E98°39'24"	2295	4.62
Yushania lineolata-MN	Z11053	V	Mianning, Sichuan	N28°27'02"	E101°49'34"	2564	6.60
Yushania lineolata-YS	wxq053	V	Yongshen, Yunnan	N26°46'50"	E100°51'25"	3089	7.29
Yushania maculata-PG	Z11049	V	Puge, Sichuan	N27°34'59"	E102°23'48"	2750	15.39
Yushania maculata-QJ	wxq099	V	Qiaojia, Yunnan	N27°05'13"	E102°59'23"	2910	12.58
Yushania polytricha-YD	wxq094	V	Yongde, Yunnan	N24°10'56"	E99°39'55"	2476	15.86

Yushania polytricha-YL	wxq067	V	Yunlong, Yunnan	N25 °36'42"	E99 °05'50"	1989	5.42
Yushania qiaojiaensis	wxq103	V	Qiaojia, Yunnan	N27 °05'01"	E103 °08'21"	3142	11.49
Yushania violascens-LP	wxq065	V	Lanping, Yunnan	N26 °42'24"	E99 °31'04"	2475	14.16
Yushania violascens-SG	wxq055	V	Shanggeri-la, Yunnan	N27 °21'59"	E99 °56'43"	2704	10.79
Yushania violascens-WX	wxq058	V	Weixi, Yunnan	N27 °28'21"	E99 °20'47"	2367	10.90
Yushania weixiensis-SG	wxq057	V	Shanggeri-la, Yunnan	N27 °59'57"	E99 °42'14"	3602	11.78
Yushania weixiensis-WX	wxq063	V	Weixi, Yunnan	N27 °11'30"	E99 °24'02"	3288	17.81
Acidosasa purpurea	wxq151	VI	Jinping, Yunnan	N22 °55'45"	E103 °16'40"	1272	14.32
Indosasa sinica	wxq120	VI	Xichou, Yunnan	N23 °21'19"	E104 °40'52"	1670	5.28
Pseudosasa amabilis	PA	VI	Guangning, Guangdong	N23 °44'52"	E112 °18'49"	267	5.74
Gaoligongshania megalothyrsa	Z11016	IX	Gongshan, Yunnan	N27 °43'39"	E98 °33'18"	2086	5.53
Outgroups							
Bonia amplexicaulis	BA		Longcheng, Guangxi	N22 °21'02"	E106 °49'24"	140	25.87
Dendrocalamus latiflorus	DL		Xichou, Yunnan	N23 °21'24"	E104 °41'51"	1020	22
Guadua angustifolia	12282		Jinghong, Yunnan	N21 °55'54"	E101 °15'12"	548	17.17

Table S2 Summary statistics of the RAD loci based on different set of minimum depth of coverage based on Stacks pipeline.

No	Voucher	Samples	m=5			m=10		
			Stacks	Depth	Utilized reads	Stacks	Depth	Utilized reads
1	wxq151	*Acidosasa purpurea*	378,437	11.9852	5,351,006	84,147	26.6357	3,271,687
2	AEM	*Bashania fangiana-EM*	442,691	23.5486	11,639,213	176,661	44.2589	9,564,314
3	AWL	*Bashania fangiana-WL*	472,935	23.526	12,060,733	211,281	40.4667	10,082,408
4	wxq149	*Bashania qiaojiaensis*	110,813	19.5721	2,864,821	18,600	75.9159	2,131,091
5	123480	*Chimonobambusa metuoensis*	237,182	15.7483	4,522,514	31,534	67.0974	3,047,502
6	wxq091	*Chimonobambusa ningnanica-BS*	176,522	13.8948	3,169,560	26,660	48.6645	2,023,489
7	wxq100	*Chimonobambusa ningnanica-QJ*	56,921	15.8315	1,208,537	7597	70.6972	817,768
8	wxq128	*Chimonocalamus fimbriatus*	71,434	14.9937	1,435,510	10,155	60.6635	958,777
9	wxq130	*Chimonocalamus pallens*	145,136	14.4472	2,671,817	23,121	50.4053	1,744,833
10	123651	*Drepanostachyum ampullare*	77,552	14.0937	1,502,920	9506	62.863	963,697
11	wxq139	*Fargeisa caduca*	56,017	13.9406	1,101,221	8510	51.0269	720,583
12	wxq106	*Fargesia aff canaliculata-QJ2*	115,589	12.548	1,754,352	17,492	42.0677	1,057,203
13	wxq098	*Fargesia aff. canaliculata-QJ1*	169,968	12.29	2,493,684	32,437	32.5172	1,511,248
14	wxq079	*Fargesia altior-LS*	174,169	16.7131	3,730,750	34,692	51.3665	2,601,837
15	wxq084	*Fargesia altior-TC*	84,095	20.8414	2,336,738	16,799	71.9577	1,750,700
16	Z11052	*Fargesia canaliculata-MN*	110,518	12.1426	1,712,673	14,372	45.5173	1,001,505
17	Z11026	*Fargesia declivis*	66,876	18.006	1,562,391	12,003	64.6085	1,143,439
18	wxq093	*Fargesia edulis-BS*	93,043	16.3342	2,017,133	15,605	59.0212	1,398,805
19	wxq059	*Fargesia edulis-WX*	132,980	20.4474	3,517,481	27,907	66.4054	2,627,776
20	wxq070	*Fargesia edulis-YL*	76,650	22.6722	2,275,944	14,862	83.5754	1,729,288
21	wxq050	*Fargesia fractiflexa-BC*	71,689	13.6701	1,327,090	11,035	48.7635	844,213
22	wxq054	*Fargesia fractiflexa-SG*	340,692	15.1457	6,098,860	119,717	27.5031	4,358,260

23	Z11014	*Fargesia melanostachys-FG*	35,082	31.4795	1,507,798	9149	95.4131	1,250,713
24	wxq056	*Fargesia melanostachys-SG*	156,499	18.8715	3,628,570	30,856	63.3283	2,657,763
25	FN	*Fargesia nitida*	276,638	18.8233	5,955,980	64,005	53.3799	4,404,721
26	wxq002	*Fargesia robusta*	32,757	24.9786	1,175,190	7595	78.9002	943,717
27	wxq126	*Ferrocalamus rimosivaginus*	78,717	14.3527	1,622,868	7659	77.8684	1,053,310
28	Z11016	*Gaoligongshania megalothyrsa*	39,547	24.4539	1,473,798	6641	99.4698	1,142,350
29	HA	*Himalayacalamus asper*	71,476	16.371	1,446,533	9221	77.0005	995,065
30	wxq129	*Indocalamus jingingensis*	53,424	18.5825	1,392,477	6428	97.4219	997,723
31	wxq120	*Indosasa sinica*	39,291	15.9979	984,474	6003	63.3072	660,530
32	PE	*Phyllostachys edulis*	453,691	13.6942	6,609,727	197,053	21.1896	4,879,916
33	wxq016	*Phyllostachys nidularia*	232,850	10.834	3,048,703	28,416	37.1215	1,606,436
34	PJ	*Pleioblastus juxianensis*	49,294	13.5513	1,032,781	6178	57.9178	643,540
35	PA	*Pseudosasa amabilis*	61,221	12.5422	1,152,753	6744	56.4125	686,487
36	SH	*Shibataea hispids*	107,122	11.3624	1,527,471	9830	54.9079	835,787
37	wxq076	*Yushania aff. levigata-LS*	46,150	19.4021	1,207,417	8581	70.0601	892,412
38	YEM	*Yushania brevipaniculata-EM*	438,381	22.8623	11,428,417	183,113	41.2821	9,380,447
39	YWL	*Yushania brevipaniculata-WL*	467,335	24.2694	12,161,654	230,484	39.3148	10,393,827
40	wxq131	*Yushania brevis*	96,515	16.5706	2,092,214	16,120	60.7372	1,474,778
41	wxq096	*Yushania levigata-YD*	209,714	17.3904	4,431,272	44,962	51.1717	3,187,313
42	Z11053	*Yushania lineolata-MN*	66,643	18.0355	1,665,940	12,430	62.8114	1,205,900
43	wxq053	*Yushania lineolata-YS*	94,410	18.0479	2,184,908	16,979	64.8317	1,567,670
44	Z11049	*Yushania maculata-PG*	326,403	15.4832	5,813,418	76,151	39.2656	4,022,070
45	wxq099	*Yushania maculata-QJ*	209,723	16.4504	4,236,449	42,786	49.109	2,959,220
46	wxq094	*Yushania polytricha-YD*	332,275	16.4718	6,209,949	97,579	35.4558	4,504,097
47	wxq067	*Yushania polytricha-YL*	69,558	17.5237	1,564,770	11,661	66.5912	1,116,324
48	wxq103	*Yushania qiaojiaensis*	170,138	17.5252	3,712,057	33,891	55.832	2,658,842

49	wxq065	*Yushania violascens-LP*	275,750	15.9345	5,279,397	73,004	36.905	3,716,554
50	wxq055	*Yushania violascens-SG*	181,978	15.9608	3,628,928	37,646	46.3688	2,531,173
51	wxq058	*Yushania violascens-WX*	222,207	14.6639	3,908,684	51,325	36.5811	2,670,616
52	wxq057	*Yushania weixiensis-SG*	221,853	17.0509	4,423,904	51,695	46.3203	3,187,920
53	wxq063	*Yushania weixiensis-WX*	325,895	18.5908	7,093,784	110,943	37.6683	5,405,602
54	BA	*Bonia amplexicaulis*	358,608	33.2713	12,351,679	308,783	37.2123	11,980,245
55	DL	*Dendrocalamus latiflorus*	496,805	19.5966	10,179,288	304,765	26.5368	8,782,083
56	12282	*Guadua angustifolia*	478,116	14.4331	7485,858	187,000	23.5051	5,423,307
Average value			186392.41	17.46	3928108.18	57506.59	54.52	2949444.30
